# Adrenal Lesions: A Review of Imaging

**DOI:** 10.3390/diagnostics12092171

**Published:** 2022-09-08

**Authors:** Benedetta Bracci, Domenico De Santis, Antonella Del Gaudio, Maria Carla Faugno, Allegra Romano, Mariarita Tarallo, Marta Zerunian, Gisella Guido, Michela Polici, Tiziano Polidori, Francesco Pucciarelli, Iolanda Matarazzo, Andrea Laghi, Damiano Caruso

**Affiliations:** 1 Department of Medical Surgical Sciences and Translational Medicine, Sapienza—University of Rome, Radiology Unit—Sant’Andrea University Hospital, 00189 Rome, Italy; 2 Department of Surgery “Pietro Valdoni”, Sapienza University of Rome, 00185 Rome, Italy

**Keywords:** adrenal, incidentaloma, adenoma, myelolipoma, computed tomography

## Abstract

Adrenal lesions are frequently incidentally diagnosed during investigations for other clinical conditions. Despite being usually benign, nonfunctioning, and silent, they can occasionally cause discomfort or be responsible for various clinical conditions due to hormonal dysregulation; therefore, their characterization is of paramount importance for establishing the best therapeutic strategy. Imaging techniques such as ultrasound, computed tomography, magnetic resonance, and PET-TC, providing anatomical and functional information, play a central role in the diagnostic workup, allowing clinicians and surgeons to choose the optimal lesion management. This review aims at providing an overview of the most encountered adrenal lesions, both benign and malignant, including describing their imaging characteristics.

## 1. Introduction

Adrenal lesions are serendipitously discovered in up to 5% of cross-sectional examinations performed for other purposes and are hence labeled incidentalomas. Adrenal lesions can be classified as benign or malignant or functioning or nonfunctioning. An adrenal lesion is defined as functioning if it overproduces of one or more hormones or their metabolites, whereas a nonfunctioning lesion is evident as an increased volume of the adrenal gland without hormonal overproduction. Both benign and malignant lesions can be functioning or nonfunctioning; however, the most common adrenal lesions in the general population are nonfunctioning benign tumors, found in approximately 5% of all abdominal computed tomographic (CT) examinations [1,2]. Once an adrenal mass is identified, a comprehensive adrenal hormone evaluation helps to define the adrenal function and the eventual type of hormonal hypersecretion.

The management of incidentaloma is mostly observational, while the identification of cortisol hypersecretion is very important due to the higher incidence of cardiovascular events in this group of patients, in particular in young female patients. This last category of patients represents a high-cardiovascular-risk group that may benefit from surgical resection of adrenal tumors [3].

Abdominal ultrasound (US) is usually performed as screening examination in patients with abdominal discomfort or nonspecific abdominal pain, symptoms potentially related to adrenal incidentalomas. Nevertheless, US detection rate is significantly lower compared with CT and magnetic resonance (MRI) in the identification of lesions smaller than 3 cm; therefore, the role of US in the identification of adrenal masses is marginal. Additionally, most adrenal nodules have nonspecific characteristics at US; therefore, this imaging modality cannot correctly distinguish between benign and malignant nodules. On the contrary, US is a reliable tool in lesion staging [4].

Adrenals are routinely seen with CT examinations of both abdominal and thoracic scans. Thus, CT is commonly the first imaging modality identifying adrenal lesions, due to its widespread use and high spatial and temporal resolution. Unenhanced acquisition allows for precise lesion measurement and accurate density evaluation. In cases of diagnostic uncertainty, a multiphasic acquisition protocol helps with achieving the correct diagnosis. CT can be also used in selected cases to guide needle biopsy for the staging and planning of oncologic treatment.

MRI also plays a pivotal role in adrenal lesion characterization owing to the high-contrast resolution and inherent tissue characterization ability, which is particularly useful in the diagnosis of adrenal adenomas and in the identification of different tissue components in cases of in homogeneous lesions.

Lastly, functional imaging might be part of the diagnostic workup in selected cases. Fluorodeoxyglucose (FDG) PET-CT combines morphological and functional imaging, aiding in the timely differentiation between benign and malign adrenal lesions. In fact, malignant lesions are usually characterized by increased tissue metabolism before anatomic alterations [5]. Adrenal FDG uptake is deemed of malignant origin when it is higher than hepatic FDG uptake. However, since the normal adrenal SUVmax range is between 0.95 and 2.46 and the liver SUVmax range ranges from 1.5 to 2.0, the overlap might lead to an incorrect diagnosis. To overcome this limitation, the adrenal/liver SUV ratio has been proposed to differentiate benign from malignant lesions [6,7]. PET-CT limitations include non-FDG-avid primary tumors and the inability to differentiate malignant lesions [7].

## 2. Benign Lesions

### 2.1. Adenoma

Adenomas are the most common among benign adrenal tumors, representing 50–80% of adrenal lesions [8]. The incidence increases with age, from 0.14% in the age range of 20–29 years up to 7% in patients over 70 years [7,9,10]. Most adenomas occur as an occasional finding; more than 75% of incidentally adrenal lesions are adenomas [11].

Adrenal adenomas’ first evaluation aims to determine if is functioning or not and its benign behavior; even though CT cannot differentiate between functional and nonfunctional adenomas, it can suggest a functioning adenoma when the contralateral adrenal gland is atrophied [11,12,13]. When functioning, their clinical presentation depends on the secreted hormone: Cushing’s syndrome in cortisol-secreting tumors, Conn’s syndrome in aldosteron-secreting tumors, and adrenal virilization in cases of androgen secretion. Roughly 20% of adenomas appear as bilateral lesions and tend to present with autonomous cortisol secretion (ACS) [14]. If the first approach is inconclusive, a multidisciplinary evaluation should be performed (nuclear or functional imaging or other endocrine tests). In addition, the latest evidence shows that texture analysis features based on CT and MRI images allow for differentiating malignant from benign adrenal lesions with good accuracy. If a suspected malignant or functioning form is found, a multidisciplinary team should plan an appropriate follow-up [15]. Moreover, recent studies reported that the inactivating variants in the ARMC5 (Amadilo repeats containing 5; OMIM 615,549) gene located on chromosome 16p11.2 are the most common genetic cause of PBMAH (primary bilateral macronodular adrenal hyperplasia), which is a rare type of Cushing syndrome characterized by the bilateral enlargement of the adrenal glands, large adrenal nodules (>10 mm), and increased cortisol production. In this context, it is very important to combine imaging examination with the study of adrenal function in patients with bilateral adenomas [16].

On baseline CT, the characterization of an adrenal lesion is based on the presence of intracellular lipids. In fact, the majority of adrenal adenomas (70% circa) contain a substantial amount of cytoplasmic fat (lipid-rich adenomas) and are easily recognizable on CT scan [17]. Attenuation values ≤10 HU are diagnostic indicators of a lipid-rich adenoma with a sensitivity of 71% and a specificity of 98% [18]. Up to 30% of adrenal lesions have attenuation values higher than 10 HU due to poor content of intracytoplasmic lipid (lipid-poor adenomas) and may be misdiagnosed as malignant. A recent study conducted by F. Ceccato and colleagues analyzed the growth and attenuation of benign adrenal incidentaloma during a CT follow-up and adrenal a tendency to decrease the mean attenuation value after 5 years of follow up that corresponded to an increase in lipid content and to a reduced cortisol suppression of 1 mg after the dexamethasone suppression test (DST). These findings suggest an interesting correlation between the variation in lipid content of adenoma and its endocrine function, which can be assessed by CT scan [19]. Lanoix et al. analyzed CT and MRI imaging of patients with atypical adenomas (adrenal lesions with heterogeneous attenuation or signal intensity, heterogeneous signal drop on CSI sequences or signs of hemorrhage, necrosis, calcifications, etc.) in order to distinguish them from non-adenoma lesions. They categorized the distribution of the microscopic fatty component of the lesion on CT or MRI as “diffuse” (in a major part of the lesion), “multiple foci” (punctiform spots among the lesion), “partial” (at least one third of the size of the lesion), or “crescent-like” (if they were thin and peripheral). Their analysis found that the diagnosis of atypical (heterogeneous) adenoma versus non-adenoma was achievable with a specificity of 98% and a sensitivity of 54% if microscopic fat was present inside a heterogeneous adrenal lesion at CT/MRI, even if partial [20].

A rapid enhancement and a rapid contrast medium washout is typical of adenomas. On the contrary, metastases are characterized by conspicuous angiogenesis and leaky capillaries, leading to prolonged contrast medium retention and consequent slower washout. A dedicated CT protocol consisting of unenhanced acquisition, portal venous phase, and 15-min delayed phase allows for the calculation of absolute and relative percentage washout (APW and RPW, respectively), resulting from the ratio between the attenuation values of the lesion on the delayed phase and the initial dynamic contrast-enhanced acquisition [17,21,22]. Specifically, APW is calculated as follows [7]:

$$\frac{HU_{portal\;venous\;phase}-HU_{delayed\;phase}}{HU_{portal\;venous\;phase}-HU_{unenhanced\;phase}}\times 100$$

RPW is obtained when no unenhanced CT scan is available, with the following formula [7]:

$$\frac{HU_{portal\;venous\;phase}-HU_{delayed\;phase}}{HU_{portal\;venous\;phase}}\times 100$$

An absolute washout greater than or equal to 60% has a sensitivity of 86–94% and a specificity of 92–96% for the diagnosis of adrenal adenoma. Accordingly, a relative washout greater than or equal to 40% reported a sensitivity of 96% and a specificity of 100% for the diagnosis of adenoma [7,23,24].

Although CT achieves good performance in characterizing adrenal adenomas, the intrinsic ability of MRI to characterize soft tissues makes it a tool as sensitive as CT in the assessment of adrenal lesion [7]. In particular, MRI overcomes CT diagnostic performance, especially when an adrenal lesion has a density between 10 and 20 HU on unenhanced CT [25]. An essential MRI feature is its ability to detect intralesional fat using chemical shift imaging (CSI) [17]. This technique takes advantage of different frequencies of precession of water and fat for each voxel to identify intracellular lipid content. In detail, fat and water have different chemical environments surrounding their protons. Electrons surrounding protons generate a shield against an external field, and since fat protons are more shielded than water protons, fat molecules are less affected by the magnetic field [26,27]. In fact, fat protons precess at a lower frequency than water protons, alternating between in and out of phase [28]. Selecting a specific echo time (TE), it is possible to obtain two different signals: in the in-phase sequences, fat and water proton signals are additive, while in the out-of-phase sequences, fat and water signal are maximally opposed (180°), resulting in signal loss [7,17,25,26,28].

**Figure 1:**
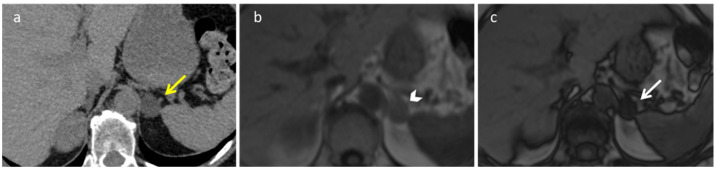
Adrenal adenoma in a 63-year-old woman. Unenhanced CT (**a**) shows a 2 cm left adrenal lipid rich adenoma (yellow arrow) characterized by attenuation values lower than 10 HU. Axial T1-weighted in-phase (**b**) and out-of-phase (**c**) MR images demonstrate a signal loss in the out-of-phase images (white arrow).

A practical and widely used diagnostic tool allowing for diagnosing a typical adrenal adenoma is a visible signal dropout in out-of-phase images compared with the in-phase sequences displayed with identical windows (Figure 1) [29].

Along with this qualitative approach, quantitative methods have been established to diagnose adrenal adenoma. In detail, the adrenal-to-spleen CSI ratio (ASR) or the adrenal signal intensity index (ASII) can be obtained by positioning regions of interest (ROIs) within the lesion in both in-phase and out-of-phase sequences and then calculating the signal drop.

ASR formula:

$$\left[\frac{\left({lesion\;}_{out\;of\;phase}\right)/\left({spleen\;}_{out\;of\;phase}\right)}{\left({lesion\;}_{in\;phase}\right)/\left({spleen\;}_{in\;phase}\right)}-1\right]\times 100;$$

ASII formula:

$$\frac{\left({lesion\;}_{in\;phase}\right)-\left({lesion\;}_{out\;of\;phase}\right)}{\left({lesion\;}_{out\;of\;phase}\right)}\times 100$$

ASR cutoff values for the diagnosis of adrenal adenoma are ≤−35.9% at 1.5 T and ≤−17.2% at 3 T. Alternatively, liver or muscle can replace the spleen in such calculations, with different cutoff values [30]. ASII cutoff values for the diagnosis of adenoma are >16.5% at 1.5 T and >1.7% at 3 T [30,31]. Diffusion-weighted imaging is not a determinant of an adrenal adenoma diagnosis: Since adrenal adenomas tend to demonstrate restricted diffusion, there are no significant differences in ADC values of adenomas and malignant lesions [7,27].

### 2.2. Myelolipoma

**Figure 2:**
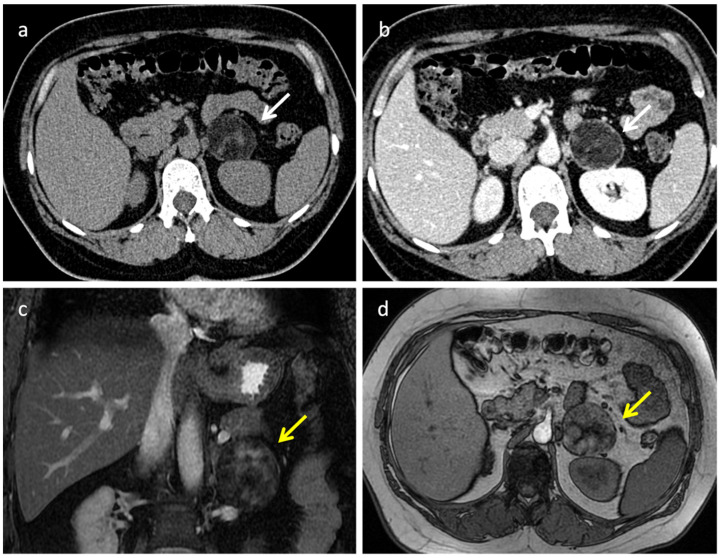
Adrenal myelolipoma in a 42-year-old woman. CT demonstrates a large mass within the left adrenal gland (white arrow in (**a**,**b**) and yellow arrows in (**c**,**d**)), characterized by well-defined borders and internal inhomogeneity. Axial unenhanced CT (**a**) shows attenuation values <−30 HU due to the presence of gross fat. On portal venous phase (**b**), the mass shows subtle enhancement due to its poor vascularization. Coronal T2 fat sat sequence (**c**) and axial T1-weighted out-phase image (**d**) demonstrate extracellular fat.

Adrenal myelolipomas (AMLs) represent 6–16% of adrenal incidentalomas, second only to adenomas [32]. They are benign adrenal masses, usually unilateral, small, and non-functional. AMLs are heterogeneous lesions composed of variable amounts of fat, myeloid cells, erythroid cells, and occasionally calcification. US has a limited role in the characterization of the adrenal masses as it provides information only regarding their composition (solid or cystic). On US, fat-predominant lesions appear hyperechoic, and when composed of myeloid elements, they may appear hypoechoic. Frequently, these lesions may present with mixed hyperechoic and hypoechoic areas due to varying amounts of fat and myeloid elements [33]. At CT, AML appears as a round mass with well-defined borders and can be easily identified by the presence of macroscopic fat (Figure 2); however, density varies depending on the proportion of fat and myeloid components [34].

On PET-CT, AML appears as a mass that shows minimum homogeneous FDG uptake in the soft tissue and no FDG uptake in the fat component [35]. On MRI, macroscopic fat components are hyperintense on T1- and T2-weighted images, while blood-containing components are hypointense on T1-weighted and moderately hyperintense on T2-weighted images. In accordance with the presence of macroscopic fat, AMLs are characterized by signal dropout in fat-suppressed T1- and T2-weighted sequences. On T1-w opposed-phase images, a characteristic India ink artifact is seen at the masses’ fat–water interfaces; this artifact is diagnostic of the presence of macroscopic fat and characteristic of AML [36]. Based on MRI features, it is possible to discriminate AMLs into three groups [37]:

- Homogeneous, distinctive of lesions predominantly composed of fat;
- Heterogeneous, distinctive of lesions composed of mixed fatty and myeloid elements;
- Nodules, mass-like areas primarily composed of myeloid cells.

As opposed to lipid-rich adenomas, AMLs contain extracellular lipids; therefore, they do not account for signal drop on out-of-phase images [29]. When AML is identified by noninvasive imaging techniques, there is no need for follow-up. Surgical removal is indicated for nodules with a diameter over 4 cm [38]. Treatment is necessary only with large lesions, as rare cases of spontaneous intralesional hemorrhage have been described, or when they become symptomatic due to pressure effects [39].

### 2.3. Adrenal Hyperplasia

**Figure 3:**
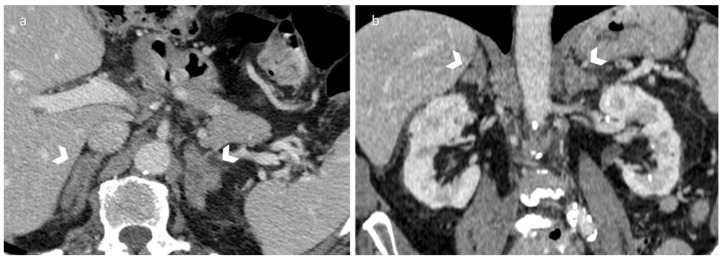
Axial unenhanced CT (**a**) and coronal reformatted portal venous phase of contrast-enhanced CT (**b**) of a 65-year-old man with bilateral adrenal hyperplasia. Note the symmetrical bilateral thickening of adrenal glands, presenting normal contours and no mass lesions.

The adrenal glands are triangular-shaped organs with an inverted Y morphology, measuring circa 5 cm by 2 cm, with a weight of 4–5 g each [22,40]. The average width of the adrenal glands is between 6 and 8 mm, with a slightly greater width of the left adrenal gland [41]. Adrenal hyperplasia is a benign condition consisting in a diffuse or focal glandular enlargement; it can be congenital or acquired over the course of life (Figure 3).

Adrenal cortical hyperplasia can be separated into two different clinical categories: ACTH-dependent Cushing’s syndrome and ACTH-independent Cushing’s syndrome [42]. ACTH-dependent syndrome includes Cushing’s disease, consisting of the production of ACTH by a pituitary adenoma or, in rare cases, by a pituitary carcinoma, and in ectopic ACTH production. The most frequent neoplastic cause of the ectopic production of ACTH are lung neoplasms, in particular bronchial carcinoids, followed by small cell lung cancer [43]. Among the ACTH-independent Cushing’s syndromes, there is primary pigmented nodular adrenal disease (PPNAD), ACTH-independent macronodular adrenal hyperplasia (AIMAH), and congenital adrenal hyperplasia (CAH). Typically, there is a homogeneous bilateral thickening of the whole gland, which appears enlarged (with an adrenal body thickness >10 mm and adrenal limb thickness >5 mm) while retaining the adreniform shape without any measurable adrenal nodules. This diffuse enlargement can take a smooth or nodular appearance, where the latter is mostly viewed in asymptomatic patients with prolonged exposure of ACTH and consists of confluent adrenal irregularities alternated by atrophic adrenal tissue [41,44]. In their study, Benitah et al. analyzed the correlation between the baseline adrenal gland’s morphology on CT examination (smoothy enlarged or nodular) with the development of adrenal metastasis in lung cancer patients without finding an increased risk. However, a hyperplastic stress response to cancer can explain why a smooth enlargement of the adrenal gland is a usual finding in patients with malignancy. An association has been found between adrenal gland nodularity and older age. In fact, small nodular adenomas are more common in older patients [45]. On MRI, enlarged hypertrophic adrenal glands are well delineated on T2-weighted sequences with fat suppression and appear hyperintense relative to surrounding fat [37]. Ultrasound is very often used in CAH, where the adrenal glands may appear enlarged and cerebriform.

The differential diagnosis of adrenal hyperplasia determines neoplastic versus non-neoplastic lesions. Among the non-neoplastic causes of adrenal hyperplasia should be considered adrenal hematomas and adrenal infections, often bilateral and generally caused by cytomegalovirus in immunosuppressed individuals [41]. Among the neoplastic lesions should be mentioned neurofibromatosis, commonly responsible for macronodular cortical hyperplasia, and lymphomas. Neurofibromas are benign nerve sheath tumors (perineural and Schwann cells) usually encountered in NF-1. Solitary neurofibromas of adrenal glands are extremely rare, and only a few cases are reported in literature. Neurofibromas are well-circumscribed, non-capsulated lesions; they can be focal or plexiform and are characterized by low CT attenuation and poor enhancement [46]. On MRI images neurofibromas show hypointensity on T1-w sequences, a heterogeneous hyperintensity on T2-w sequences with “fascicular sign” and areas of low T2-w signal intensity due to the presence of collagen and fibrous tissue that enhances after contrast medium administration [47]. Primary adrenal lymphoma is bilateral in 50% of cases; on CT scans, it appears as a single complex hypoattenuating lesion with slight post-contrast enhancement and no calcification. In secondary adrenal lymphoma, the adrenal glands have an increased size but a more homogeneous morphology than primary lesions. On MRI, adrenal lymphomas have nonspecific features: low/intermediate signal intensity in T1-weighted images and moderately high signal intensity in T2-weighted images, and after contrast medium injection, they appear hypovascular compared with normal parenchyma [48].

New evidence focuses on the new surgical approach, which tends to favor unilateral adrenalectomy and avoid bilateral procedures in patients with bilateral adrenal hyperplasia with hypercortisolism. In fact, bilateral adrenalectomy leads to definitive adrenal insufficiency, which exposes patients to increased cardiovascular diseases and infections and to lifelong hormone replacement. This is the reason unilateral adrenalectomy could be an interesting option in selected, mostly young, patients with PPNAD, basing the selection of the side of the adrenalectomy on the eventual presence of a macronodule or an asymmetry of the uptake on 131 I-norcholesterol [49].

### 2.4. Cyst

Adrenal cysts are relatively rare, with a prevalence up to 0.2% and a marked female predilection, with a male-to-female ratio of 1:3 [50]. They are commonly unilateral and occur as incidental findings, as they tend to be nonfunctional. They are characterized by a minimal enlargement over time, with a median size change of 6 mm and a median growth rate of 2 mm/year [51]. When symptomatic, they generally occur with abdominal pain and hypertension. In cases of large functional lesions, malignancy should be suspected [8]. Adrenal cysts derive from epithelial or endothelial proliferation, hemorrhage, or parasitic disease. Cysts can be classified in four categories, namely, endothelial (over 80%), epithelial, parasitic, and pseudocysts; the latter are normally considered a sequela of prior hemorrhage or trauma [40].

**Figure 4:**
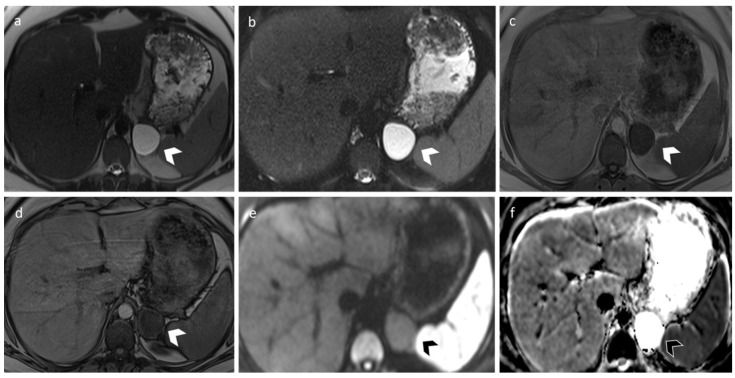
A 33-year-old female with an incidental left adrenal cyst. MR revealed a 4 cm nodular lesion in the left adrenal gland characterized by thin walls and high and homogeneous signal in T2 (**a**) and T2 fat-sat sequences (**b**). T1 signal (**c**,**d**) is in accordance with simple fluid content, and DWI/ADC sequence (**e**,**f**) does not show signs of hypercellularity.

Adrenal cysts are relatively easy to characterize on radiological examinations. On US, endothelial cysts generally appear as well-circumscribed thin-walled lesions, with anechoic or hypoechoic content. In cases of previous hemorrhages, fluid–fluid levers or debris may be present. On CT examination, they appear as well-circumscribed round masses, with homogenous density close to that of water (<20 HU) and thin walls. Increased attenuation might be a sign of hemorrhage, and calcifications can also be present, either mural or scattered within the cyst. On MRI scans, uncomplicated adrenal cysts show hyperintensity on T2-weighted images, low signal on T1-weighted images, and no contrast enhancement. T1-hyperintense content is a usually secondary to hemorrhage (Figure 4) [34].

### 2.5. Schwannoma

Schwannomas are extremely rare tumors, commonly benign, originating from the nerve sheath. Fewer than 40 cases of adrenal schwannomas have been reported. They are commonly asymptomatic and present as incidental findings; nevertheless, patients with lesions larger than 5 cm may experience minor symptoms, such as abdominal pain and discomfort, probably due to mass effect.

At CT scans, they appear as well-circumscribed, hypodense lesions; larger lesions may undergo degenerative changes, such as cystic areas, calcifications, or hemorrhagic components. On MRI examination, adrenal schwannomas tend to have low signal intensity on T1-weighted images and inhomogeneous hyperintense on T2-weighted images, due to the coexistence of compact cellular areas and paucicellular zones. This structural characteristic accounts for the heterogeneous enhancement after contrast media injection [52].

## 3. Malignant Lesions

### 3.1. Pheochromocytoma

Pheochromocytoma (PCC) is a tumor arising from the adrenal medulla. PCCs follow the 10% rule: 10% of cases are bilateral, 10% occur in children, 10% are non-secreting, 10% are bilateral, 10% are malignant, 10% are asymptomatic, and 10% arise outside of the adrenal gland from the sympathetic chain (paraganglioma). PCCs are neuroendocrine tumors originating from chromaffin cells and secreting catecholamines; typical symptoms include hypertension, tachycardia, sweating, flushing, hyperglycemia, headache, and anxiety [28]. These lesions can be associated with multiple syndromes: neurofibromatosis type 1, von Hippel-Lindau disease, Carney’s complex, and multiple endocrine neoplasms (MEN) 2A and 2B [53]. In patients with suspected PCC, it is first necessary to perform laboratory tests such as 24-h urine vanillylmandelic acid, urinary and plasma metanephrines measurements [54]. PCCs are functional tumors and are usually of small size at diagnosis [17].

**Figure 5:**
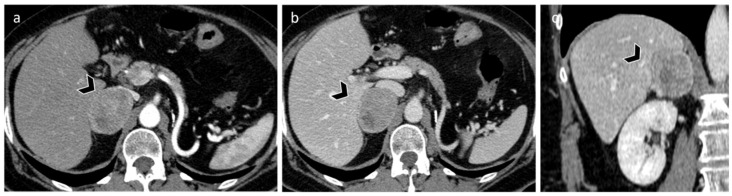
A 65-year-old female with right adrenal pheochromocytoma. CT axial arterial (**a**) and portal venous phases (**b**,**c**) show inhomogeneous adrenal solid lesion with strong and heterogeneous enhancement; the lesion showed an absolute wash-out of 77%.

On US, they appear encapsulated and hypervascular on echo color Doppler, showing early enhancement on contrast enhanced ultrasound sonography (CEUS) [55]. On unenhanced CT scans, PCC has no specific characteristics, although attenuation values >150 HU are suspicious for this type of lesion [56]. Arterial phase shows avid contrast enhancement, with 75% having slow washout, less than 60% absolute washout, and less than 40% relative washout. Additionally, 25% of PCCs might have similarities to adenomas, especially the small ones; some characteristics such as necrosis, heterogeneous enhancement, and higher attenuation are more indicative of PCC than adenomas (Figure 5) [57].

**Figure 6:**
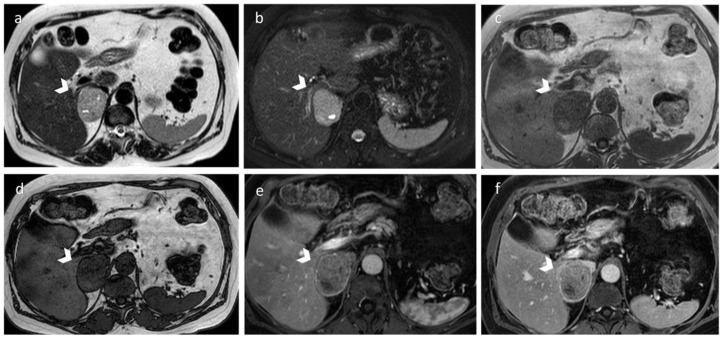
A 65-year-old female with right adrenal pheochromocytoma. MRI revealed a 5 cm solid lesion characterized by intermediate T2 signal (**a**,**b**) and no T1 signal drop in T1 out-of-phase sequences (**c**,**d**). Post-contrast acquisitions in arterial and portal venous phase (**e**,**f**) reveal inhomogeneous signal intensity and necrotic components.

MRI has great sensitivity in the detection and characterization of PCCs, up to 98% [58]. The MR signal is slightly hypointense in T1-weighted and brightly hyperintense in T2-weighted images, with the characteristic sign of the “lightbulb bright”; however, this sign may not be present in 30% of PCCs. PCCs do not contain cytoplasmic lipids, and therefore, no signal drop is seen on out-of-phase images, unlike lipid-rich adenomas (Figure 6) [7]. PCCs can appear heterogenous on CT or MRI due to the presence of hemorrhage or necrosis [28].

MIBG scintigraphy is a nuclear medicine examination that has been used to localize PCCs and has both high sensitivity (95–100%) and high specificity (100%) [59]. It is particularly useful in cases of family history of PCC or hereditary disorders, in the localization of distant metastases, and in patients with negative CT/MRI but positive biochemical markers. Additionally, MIBG scintigraphy outperforms PET-CT in detection of PCCs, especially in case of benign lesions [58]; nevertheless, this imaging technique might lead to false negatives in cases of lesions smaller than their spatial resolutions and in the presence of necrosis and interfering drugs, ultimately responsible for absent MIBG uptake [60]. In-111 octreotide can be used as a radiopharmaceutical to detect PCC, with sensitivity of 75–90%. Abdominal imaging is performed 4 and 24 h after injection [26]. Some studies have shown that malignant pheochromocytomas on PET-CT show a greater uptake compared with benign lesions. In comparison between MIBG scintigraphy and PET-CT, although MIBG scintigraphy has greater sensitivity (88% vs. 82%), PET-CT is very useful for recognizing PCCs that do not concentrate MIBG [7]. First-choice treatment is surgical resection, with preoperative premedication with α-blockers, β-blockers, metyrosine, and volume expander, to avoid hypertensive crisis [61].

### 3.2. Metastasis

Despite their small size, adrenal glands are the fourth most common metastatic site [62]: 25% of patients with cancer are found on autopsy to have adrenal metastases [63]. Primary cancers with predilection for adrenal metastases originate from the lung, breast, melanoma, gastrointestinal tract, pancreas, and kidney [14]. Rarely, adrenal metastasis can be the first presentation of occult malignancy [21]. At least half of patients with pathologically proven adrenal metastasis had bilateral localization, but unilateral involvement may occur [28]. In patients with primary malignancy, the diagnosis of bilateral adrenal metastases is usually made by imaging with a certain degree of certainty, with no need for further biopsy and pathology confirmation. Patients with bilateral adrenal metastasis have an increased risk of adrenal insufficiency, and hence, careful clinical monitoring is required [14].

**Figure 7:**
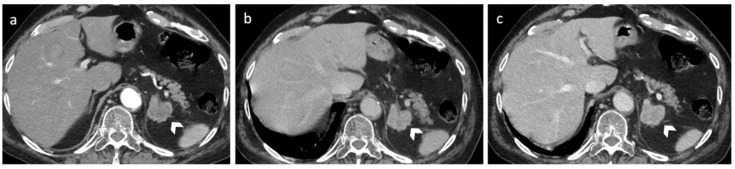
A 67-year-old female with metastatic lung cancer. Axial arterial (**a**), portal (**b**), and delayed phase (**c**) showing large, inhomogeneous, left solid 4 cm adrenal lesion with irregular margins and foci of low attenuation, in keeping with partial necrotic phenomena. No infiltration of surrounding structure is seen. The lesion was referred to as adrenal metastasis.

Ultrasound (US) is not indicated in the study of adrenal metastasis; however, it can be used in differentiating benign lesions from suspicious ones [21]. Adrenal metastases have no definite imaging findings on CT or MRI. On unenhanced CT, they commonly have attenuation values >10 HU, with irregular margins, areas of hemorrhage, and calcifications. After contrast medium injection, irregular peripheral enhancement is noted [28]. Typically, hypervascular metastases can show washout characteristics similar to adenomas (Figure 7).

**Figure 8:**
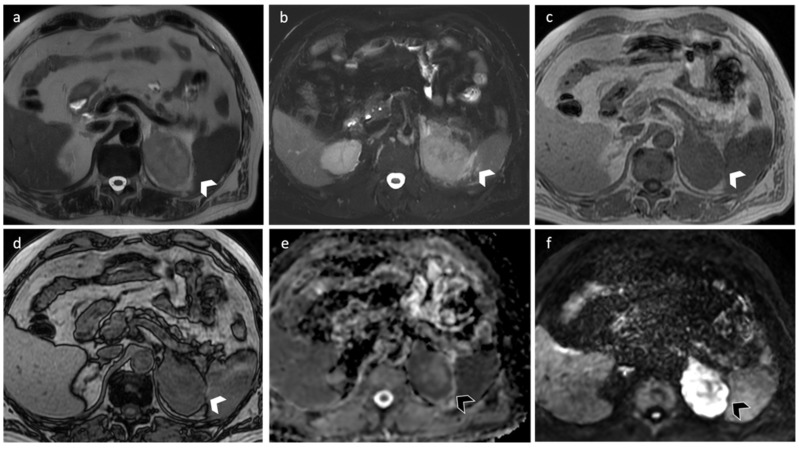
A 67-year-old man with lung cancer. MRI shows a 7 cm nodular lesion on the left adrenal gland characterized by intermediate signal intensity on axial T2-weighted images (**a**) and perilesional edema (**b**). The lesion shows intermediate T1-intensity (**c**) with no signal loss in out-of-phase sequences (**d**), due to the absence of intralesional fat. DWI (**e**) and ADC map (**f**) demonstrated hypercellularity.

On MRI, adrenal metastases usually appear hypointense on T1-weighed imaging and hyperintense on T2-weighed imaging, with cystic change and necrosis; they may show ring or uneven enhancement after contrast administration (Figure 8) [37].

It must be mentioned that metastases from clear cell renal cell carcinoma and hepatocellular carcinoma might contain intracellular fat; therefore, in such cases, CSI has limitations in distinguishing them from adenomas [21]. The diagnostic power of PET-CT in the detection of adrenal metastasis is limited. On the contrary, various adrenal lesions, namely adrenal cortical hyperplasia, adenomas, and endothelial cysts, may turn positive on PET-CT and be erroneously interpreted as metastases. Furthermore, metastasis detection at PET-CT mainly depends on the type of primary tumor and metastasis size; for instance, PET scanners have a spatial resolution of 5 mm, and metastases from non FDG-avid tumors, such as neuroendocrine tumors and pulmonary bronchoalveolar carcinoma, may not be detected on PET-CT [7]. In conclusion, the detection of adrenal metastases in patients with primary malignancy is denotative of advanced stage; it is therefore crucial to differentiate them from other lesions, such as adenomas, to outline the most appropriate clinical stage and treatment plan [58]. The surgical resection of adrenal metastasis may be performed, and it was shown to extend long-term survival in some cases [64].

### 3.3. Carcinoma

Adrenocortical carcinoma (ACC) is a rare aggressive tumor presenting in 0.7–2.0 cases/million habitants/year [65]. ACC is commonly sporadic; however, it can be associated with hereditary syndromes. Age distribution is bimodal, with a peak in early childhood and another in middle adulthood (40–50 years) [66]. Most ACCs are secretory, accounting for an overproduction of glucocorticoids or androgens; however, approximately 15% of patients with ACC may be initially diagnosed incidentally. These lesions can be fatal if left untreated, and it is therefore suggested to consider ACC in any incidental adrenal findings, especially in patients without history of malignancy [64]. In comparison with adrenal adenomas, ACC are generally larger; present with irregular margins, internal heterogeneity, and cystic degeneration; and can show areas of calcification; venous infiltration, such as invasion of the inferior vena cava, is typical for ACC [65]. Metastases are seen in approximately 30% of patients [67].

US can incidentally diagnose ACCs; these lesions present as large masses, usually over 6 cm in size, with areas of necrosis, hemorrhage, and calcifications, and are hypervascular on color Doppler [68].

On contrast-enhanced CT, ACCs show heterogeneous enhancement (peripheral or rim-like) due to areas of hemorrhage or central necrosis. Sporadically they can be smaller in size (<5 cm) and regularly shaped, especially in children, and therefore they can be mistaken for adenomas [69]. Compared with adenomas, these lesions show less absolute and relative washout; nonetheless, washout calculations are not commonly indicated in the evaluation of ACCs due to their size and heterogeneity. ACCs can infrequently show washout characteristics compatible with adenomas, and in such cases, heterogeneous enhancement and size (>4 cm) help in establishing the correct diagnosis [70]. On MRI, ACCs show heterogeneous signal, generally isointense to the liver on T1-weighted imaging and hyperintense on T2-weighted imaging. In the presence of intralesional hemorrhage, there may be a high-intensity signal on T1-weighted sequences [7]. After contrast medium injection, ACCs are characterized by heterogeneous enhancement and slow washout. In comparison with CT, MRI can better evaluate inferior vena cava invasion and neoplastic thrombosis [71,72].

ACCs show an increased uptake of FDG due to increased glucose utilization. In recent studies, PET-CT was found to be useful for chemotherapeutic response assessment as it can predict responses earlier than the detection of anatomic changes on CT. Nevertheless, PET-CT is not routinely included in the management algorithms of adrenal malignancies [73]. Surgical resection, either curative or debulking, is the treatment of choice for ACCs, regardless of tumor size and local invasion [74].

Imaging features of the aforementioned adrenal lesions are summarized in Table 1.

## 4. Conclusions

Cross-sectional imaging plays an important role in differentiating between benign and malignant adrenal lesions, and it is often decisive for their characterization, as happens with CT and MRI imaging for adenomas. Incidentaloma is a frequent finding, and it fundamentally includes benign and nonfunctioning adenomas. However, most adrenal lesions are difficult to characterize with certainty, and a clinical framework is necessary to avoid misdiagnosis, especially in oncologic patients.

## Figures and Tables

**Table 1:** Main imaging features of adrenal lesions.

Lesion	NCCT	MRI	Enhancement	Calcification	Hemorrhage	Necrosis
**Adenoma**	<10 HU	Loss of signal OP	RPW/APW ≥ 40/60%	−	−	−
**Myelolipoma**	<−30 HU	Loss of hyperintensity on T1-w fs	+/−	−	−	−
**Hyperplasia**	Depends on etiology	Depends on etiology	+/−	+/−	+/−	−
**Cyst**	Fluid	Hyper on T2-w Hypo on T1-w	−	−	+/−	−
**Schwannomas**	Hypodense	Iso on T1-w Iso/Hyper on T2-w	Inhomogeneous	+/−	+/−	−
**Pheochromocytoma**	40–50 HU	Iso/Hypo on T1-w Hyper on T2-w	++	+	+	+
**Metastasis**	>10 HU	Hypo on T1-w Iso/Hype on T2w	+/−	+	+	+
**Carcinoma**	Heterogeneous	Hypo on T1-w Hyper on T2-w	Inhomogeneous, slow wash-out; RPW/APW < 40/60%	+	+	+

APW: Absolute Percentage Washout; HU: Hounsfield Unit; MRI: Magnetic Resonance Imaging; NCCT: Non-Contrast CT; OP: out-of-phase; RPW: Relative Percentage Washout.

## Data Availability

Not applicable.
